# Symptoms of Internet Gaming Disorder in Youth: Predictors and Comorbidity

**DOI:** 10.1007/s10802-018-0422-x

**Published:** 2018-04-05

**Authors:** Lars Wichstrøm, Frode Stenseng, Jay Belsky, Tilmann von Soest, Beate Wold Hygen

**Affiliations:** 1 Department of Psychology, NTNU Norwegian University of Science and Technology, N-7941 Trondheim, Norway; 2 Department of Child and Adolescent Psychiatry, St. Olavs Hospital, Trondheim, Norway; 3 NTNU Social Science, Trondheim, Norway; 4 Department of Education and Lifelong Learning, NTNU Norwegian University of Science and Technology, Trondheim, Norway; 5 Department of Human Ecology, University of California Davis, Davis, CA USA; 6 Department of Psychology, University of Oslo, Oslo, Norway

**Keywords:** Comorbidity, Emotion regulation, Internet gaming disorder, Longitudinal, Social skills

## Abstract

Internet gaming disorder (IGD) was included in the Addendum to DSM-5 as a condition for further study. Studies of community samples using a diagnostic interview are lacking, and evaluations of the proposed symptoms, comorbidities, and predictors of IGD are scarce. To provide such information participants in a Norwegian prospective community study were assessed with a clinical interview at age 10 years. Symptoms of other psychiatric disorders were measured with the Child and Adolescent Psychiatric Assessment at ages 8 and 10 (*n* = 740). Children, parents, and teachers provided information on demographics, temperament, intelligence, executive functions, self-concept, social skills, victimization, emotion regulation, family climate, and parenting. Results indicated that IGD was present in 1.7% (95% confidence interval, 0.7–2.7) of the participants (3.0% boys and 0.5% girls). Factor analysis revealed two factors: heavy involvement and negative consequences. The positive predictive value of withdrawal, tolerance, and unsuccessful attempts to control gaming symptoms to the disorder was low. Symptoms of other common disorders correlated weakly with IGD-symptoms (i.e., from *r* = 0.07 to *r* = 0.15). Upon adjusting for gender and gaming at age 8, only limited social and emotion regulation skills at age 8 predicted more age-10 IGD symptoms. In conclusion, IGD is already present in a small percentage of Norwegian 10-year olds. At least three of the proposed symptoms -- withdrawal, tolerance and unsuccessful attempts to control gaming -- merit further study given their weak associations with the disorder. Symptoms of IGD are only marginally associated with symptoms of other psychiatric disorders and only predicted by social skills and emotion regulation deficits.

**Electronic supplementary material:**

The online version of this article (10.1007/s10802-018-0422-x) contains supplementary material, which is available to authorized users.

Electronic gaming has become an integral part of young people’s lives during the past few decades. Increasing time is spent on this activity by children and youth (Rehbein et al. 2015), raising the possibility that gaming may develop into a disorder for some (Petry and O'Brien 2013). In consequence, Internet Gaming Disorder (IGD) was recently included as a condition for further study in the 5th edition of the Diagnostic and Statistical Manual of Mental Disorders (DSM-5), and defined as “*Persistent and recurrent use of the Internet to engage in games, often with other players, leading to clinically significant impairment or distress…”*(American Psychiatric Association 2013, p. 795)*.* It remains to be examined what kind of disorder IGD really is, if any; a compulsion, an impulse control disorder, or a behavioral addiction. However, the DSM-5 substance use disorder work group noticed many similarities between pathological Internet gaming and addiction in the literature (Petry et al. 2014). Thus, drawing on the criteria for the other behavioral addiction in the DSM-5 (i.e., gambling disorder) and substance use disorders, nine symptoms were proposed (preoccupation, withdrawal symptoms, tolerance, unsuccessful attempts to control, loss of interest in previous hobbies/entertainments, continued use despite known problems, deception of others, gaming to escape negative mood, and jeopardizing friendships/education/jobs), of which at least five were needed for a diagnosis. As a newly recognized condition, there is limited information on prevalence, suitability of suggested symptoms, comorbidity, and risk and protective factors. Such information is necessary to evaluate whether IGD should be recognized in the future as a formal DSM disorder. We therefore report such information here.

## The Need for Clinical Interviews

With one exception (Ko et al. 2014) research to date has relied upon self-completed questionnaires to assess IGD or similar conditions (Lemmens et al. 2015; Rehbein et al. 2015; Young 1998). Whatever the utility and even benefits of such an approach, most screened positives for a psychiatric disorder do not fulfil diagnostic criteria when assessed by psychiatric interview (Kessler et al. 2003). Furthermore, many true positives are screened negative (He et al. 2013; Sveen et al. 2013), especially when prevalence is low, which is expected in the case for IGD (Petry et al. 2015). We have therefore reasons to believe that information derived from questionnaire studies of IGD do not necessarily generalize to IGD as assessed through a psychiatric interview. One study used a questionnaire-derived interview to assess “Internet addiction” while tapping DSM-5 critera for IGD (Ko et al. 2014), but the interview has not been described in detail. Thus, there is a need to develop a psychiatric interview to examine IGD according to DSM-5 (Petry et al. 2015). Because IGD is seldom assessed without considering comorbidity, a structured interview complying with the format of existing psychiatric interviews would be of particular interest.

## Suitability of Suggested Symptoms

There is some concern that the nine proposed IGD-symptoms in DSM-5 may be too unspecific, thereby identifying individuals with a strong interest in and devotion to Internet gaming, yet who are not addicted, evincing little or no impairment (Griffiths et al. 2016; Kardefelt-Winther 2015). Ko et al. (2014) compared a group of Taiwanese university students with IGD with a group of university students with very little Internet gaming. To decide whether the subjects had IGD or not they applied a diagnostic interview for Internet addiction which they adapted to tap Internet gaming, while also assessing all symptoms according to the DSM-5 criteria by interview. The usefulness of the proposed DSM-5 symptoms was thereafter evaluated by comparing DSM-5 criteria with diagnoses set according to the adapted Internet-addiction interview. With the exception of ‘deception’ – and to some extent ‘escape’, the nine symptoms had high sensitivity and specificity.

High sensitivity and specificity was also found for most symptoms in a questionnaire study of the general adolescent and young adult population in the Netherlands. As in the Taiwan study the ‘escape’ criterion showed diminished specificity, whereas ‘deception’ turned out to identify those with IGD with high accuracy (Lemmens et al. 2015). In even greater contrast, a questionnaire study of German adolescents found that many of the suggested symptoms in the DSM-5 (i.e., unsuccessful attempts to control, continued use despite known problems, gaming to escape negative mood, and jeopardizing friendships/education/jobs), did not add to the probability of receiving a diagnosis (Rehbein et al. 2015). There is thus considerable uncertainty with respect to the importance of individual symptoms for a diagnosis, especially when assessed through a clinical interview.

## Dimensionality

The DSM-5 conceptualizes IGD as a homogenous disorder with no subtypes. In support of such a homogenous view of IGD several factor analytic studies find that questionnaires tap one dimension (Fuster et al. 2016; Lemmens et al. 2015; Monacis et al. 2016). Others report two dimensions, typically with one tapping addictive-type behavior and the other devotion/heavy involvement in gaming (Brunborg et al. 2015; Chamarro et al. 2014; Charlton and Danforth 2007). Because all these studies relied upon questionnaires, we have no information about the dimensionality of interview-assessed DSM-5 defined IGD.

## Comorbidities, Correlates and Predictors

Symptoms of IGD co-occur with the most prevalent psychiatric disorders in childhood and adolescence, or symptoms of such disorders, including attention-deficit/hyperactivity disorder (ADHD), conduct disorders, depression, and anxiety (Kim et al. 2016; Kuss et al. 2014; Mihara and Higuchi 2017). Given that there is little symptom overlap with other common disorders in childhood and adolescence, also including impulse control disorders and ADHD, co-occurrences are likely to have other reasons than shared symptoms of IGD with other mental disorders (Angold et al. 1999; Wichstrøm et al. 2018). Co-occurrence is therefore more likely a result of causal mechanisms, as IGD may influence or be influenced by other disorders, or confounding factors that influence both IGD and other disorders. Notably, all IGD studies of comorbidity have relied on questionnaires. Thus, assessment of co-occurring symptoms of disorders as defined by the DSM system and by means of clinical interviews is lacking.

Etiological models of IGD and other potential Internet addictions are just beginning to emerge (Brand et al. 2016; Dong and Potenza 2014; Young and Brand 2017). Despite some differences, several important communalities exist between these models, which inform the present research. First, a range of core personal characteristics are hypothesized to predispose the youngster to IGD, including psychopathology, personality factors (e.g., low self-esteem, high impulsivity and negative affectivity), and a set of social cognitions (e.g., perceived loneliness, poor social competence) (Mihara and Higuchi 2017). Second, impaired cognitive attentional and behavioral control may increase the risk IGD through lack of suppression of gaming urges relative to long term goals (Dong and Potenza 2014; Wei et al. 2017), but impaired executive control may also result from heavy gaming and thus enter into a vicious circle, maintaining or even aggravating IGD (Brand et al. 2016; Dong and Potenza 2014). Third, dysfunctional coping styles with respect to everyday life stressors may lead some children to downregulate their negative emotions by gaming (Brand et al. 2016). Hence, children with poor emotion regulations skills would be particularly prone to IGD The prevailing models of IGD focus on the individual; cognitions, motives, urges, personality, and perceptions. However, gaming – and its risk and protective factors -- occurs in a social context, perhaps particularly evident in youth and adolescents, and demographics and social relations may predispose to, limit or reinforce IGD. We therefore suggest that comprehensive models of IGD should include (i) demographic, (ii) parent and (iii) peer factors. Numerous correlates of IGD and IGD-like symptoms have been identified in a recent review. Apart from those intrinsic to the games (e.g., type of games, playing online versus offline), these include three levels (i) demographic and parental/family factors, (ii) player characteristics, and (iii) social and peer factors. Concerning demographics, across ages and cultures, boys display considerably more IGD symptoms than girls, those not growing up in two-parent families evince more problematic gaming, as do children in families with high conflict or discord (Mihara and Higuchi 2017). Concerning parental factors, some researchers have drawn attention to the importance of parental monitoring, attitudes and rules about gaming (Bonnaire and Phan 2017; Martins et al. 2017; Su et al. 2018). As regards peer factors, youth with problematic gaming are generally more often victimized by bullying, have school problems, and poorer social competence than peers low on problematic gaming (Mihara and Higuchi 2017). We therefore suggest that comprehensive models of IGD should focus on demographic, parent, and peer factors in addition to individual characteristics. Other research indicate that those with IGD may have problems regulating their emotions .

Because most of the just-listed correlates and alleged predictors may equally well be consequences of IGD, prospective data are needed to discern true risk and protective factors from putative ones. A limited set of questionnaire-based longitudinal surveys have examined demographic, youth, family, and social factors that predict future problems resembling IGD, including, for example, problematic video game use (Gentile et al. 2011; Yu et al. 2015), pathological video-gaming (Haagsma et al. 2013; Liau et al. 2015), and video game addiction (Henchoz et al. 2016; Rehbein and Baier 2013). Many of these correlates have also been identified as predictors of prospective IGD-type behavior, including male gender, previous gaming, living in a single-parent family, impulsivity, conduct problems, low level of sports involvement, limited social competence, and poor self-esteem. However, to what extent such risk factors are also predictive of DSM-5 defined IGD-symptoms assessed through a clinical interview is yet unknown.

Interventions to reduce IGD may be more successful if prevention rather than remediation is the goal. The fact that gaming time increases considerably from the early school years to middle childhood (Norwegian Media Authority 2016; Pujol et al. 2016) coupled with strong indications that symptoms of IGD are indeed present already in middle childhood (Choo et al. 2015; Gentile et al. 2011), led us focus on this period given the prospect of informing preventative efforts. Drawing on a longitudinal community sample of Norwegian 10-year olds examined with a newly developed clinical interview to assess DSM-5 defined IGD, we present data on (1) IGD prevalence and symptoms, (2) the importance of specific symptoms, (3) the extent to which symptoms reflect one underlying dimension, (4) comorbidity with symptoms of DSM-IV defined disorders, and (5) age-8 cognitive, regulative, social, psychiatric, family predictors, and temperamental (measured at age 6) predictors of age-10 IGD symptoms. To the extent that previous findings on older children and adolescents also apply to younger children, and that results obtained through questionnaires also apply to interview-measured IGD, we hypothesize that more symptoms of IGD at age 10 would be predicted male gender and not living with both biological parents, and prior levels of the following: poor family climate, inconsistent parental discipline, low parental monitoring, high child temperamental negative affect and surgency, low effortful control and executive functioning, comparatively low self-esteem, social competence and emotion regulation skills, limited physical activity and sports involvement, and extensive prior gaming. We also examine the predictive power of parental socioeconomic status (SES; job level, education, income), child intelligence, and symptoms of psychiatric disorders – but advance no hypotheses regarding these factors.

## Method

### Participants and Procedure

The Trondheim Early Secure Study (TESS) comprises members of the 2003 and 2004 birth cohorts in Trondheim, Norway (*N* = 3456) (Wichstrøm et al. 2012). A letter of invitation together with a screen for emotional and behavioral problems, the Strengths and Difficulties Questionnaire (SDQ) 4–16 version (Crone et al. 2008), was sent to the parents of all children in the two birth cohorts prior the routine health check-up at age four of the children (*n* = 3358). The health nurses at the well-child clinics recruited participants. The nurses missed asking parents of 166 children, and 176 parents were excluded due to lack of proficiency in Norwegian. The study was approved by the Regional Committee for Medical and Health Research Ethics Mid-Norway and written consent was obtained. To increase variability and thus statistical power we oversampled children with higher SDQ scores. To accomplish this, children were allocated to four strata according to their SDQ scores (cut-offs: 0–4, 5–8, 9–11, and 12–40). In all, 1250 of the consenting children were drawn into the study using a random number generator with increasing probability of selection with increasing SDQ scores (0.37, 0.48, 0.70, and 0.89 in the four strata, respectively). Of the 1250 children from the four strata randomly recruited into the Study, 995 were successfully enrolled at Time 1. This sample, adjusted for stratification, was not significantly different from those who consented with respect to gender or their SDQ score (Wichstrøm et al. 2012). Statistics Norway compared the sample with available register information on all parents of 4-year olds in Trondheim in the years 2007 and 2008; the educational level of the TESS sample was virtually identical to the population’s level. The population of Trondheim is similar to the national average on several key indicators, including, average gross income per inhabitant (i.e., 99.5% of the national average), employment rate (i.e., identical to the national rate), and proportion of two-parent families (i.e., 80.0% for Trondheim and national average of 81.4%) (Statistics Norway 2010).

Children were reassessed biannually, affording investigation of 740 with valid data from the third (*n* = 699, 8.8 years, *SD* = 0.24) or fourth (*n* = 702, 10.5 years, *SD* = 0.16) assessment. Note that 43 youth did participate at the 10-year assessment did not participate at the 8-year (third) assessment. Attrition from the 8-year to the 10-year assessment was selective according to several child characteristics: symptoms of conduct disorder, *OR* = 1.55, *p* = 0.018; oppositional defiant disorder, *OR* = 1.38, *p* < 0.001; ADHD, *OR* = 1.16, *p* < 0.001; male gender, *OR* = 1.84, *p* = 0.049; problems with executive functioning, *OR* = 1.02, *p* = 0.001; low scores on word comprehension, *OR* = 0.93, *p* = 0.001; and being bullied, *OR* = 1.14, *p* = 0.039. Even so, the combined effect of these predictors on attrition was small (Cox & Snell R^2^ = 0.05). The interviewing with respect to IGD started in September 2013 and ended in June 2015. The teacher who knew the child also completed a questionnaire about the child. Table 1 presents characteristics of the TESS participants. At ages 8 and 10 children used medication for mental health problems, mostly for ADHD (*n* = 8). All Norwegian children are expected to have access to a computer and the Internet, as most homework and other assignments are turned in on the Internet. As a result 100% of all families with children report to have Internet access in Norway (Statistics Norway 2017).

**Table 1:** Descriptives of participants and study variables

Characteristics of participants at child age 10	N (Percentage)	
Ethnicity of biological mother		
Norwegian	650 (87.8)	
Other Caucasian	29 (4.3)	
South American	2 (0.2)	
African	2 (0.2)	
Asian	18 (2.4)	
Missing	39 (5.3)	
Child gender		
Boy	360 (48.6)	
Girl	380 (51.4)	
On medication for mental health problems	10 (1.4)	
Descriptives of study variables at child age 8 (possible range)	Mean	SD
Informant parent’s education (1–11)	6.91	2.68
Parental job level (1–6)	4.31	1.55
Parental gross annual income (0–13)	10.33	3.63
Parents married or cohabitating >6 months	0.77	0.42
Poor family climate (1–4)	1.44	0.61
Parental poor monitoring (1–5)	1.47	0.72
Parental inconsistent discipline (1–5)	1.64	0.79
Internet gaming time – hours per day	0.61	0.65
Child temperament – Negative affect (1–7)	3.45	1.10
Child temperament – Surgency (1–7)	3.99	1.27
Child temperament – Effortful control (1–7)	4.82	1.41
Child self-esteem (8–40)	32.57	10.65
Child social competence (0–90)	48.45	27.70
Child emotion regulation (1–4)	2.77	1.40
Child bullied, number of times previous 3 months	5.49	3.30
Child hours in moderate and vigorous physical activity per day	0.95	1.21
Child, number of training session in organized sports per week	1.77	1.41
Child intelligence – word comprehension	25.36	9.80
Child intelligence – matrices	16.51	7.88
Child poor executive function (0–146)	84.00	19.74
Child symptoms of ADHD (0–18)	1.12	2.33
Child symptoms of ODD (0–8)	0.90	1.18
Child symptoms of CD (0–15)	0.28	0.58
Child symptoms of major depressive disorder (0–9)	0.43	0.78
Child symptoms of anxiety disorders (0–21)	0.83	1.23

### Measures

#### Internet Gaming Disorder

When the data collection started in 2013 there was no available interview for IGD. We therefore developed an Internet Gaming Disorder Interview (IGDI) to assess DSM-5 defined symptoms of IGD. The complete interview guide (eText) and a short version of the criteria used (eTable 1) are presented in online Supplementary material. Procedures similar to those of the Child and Adolescent Psychiatric Assessment (CAPA; (Angold and Costello 2000) were used. The IGDI is an interviewer-based psychiatric interview of youth, which implies that the interviewer poses mandatory and optional follow-up questions and probes until enough information is obtained to decide whether a symptom is present during the previous 12 months. We chose not to interview the parents for two main reasons: First, deceiving others, perhaps most notably parents, with respect to the amount played is common among children and adolescents with problematic gaming (Ko et al. 2014; Rehbein et al. 2015). Parents might therefore underestimate the extent of gaming, and hence also the problems attributed to it. Second, many of the symptoms of IGD refer to inner states of the youth (e.g., anticipating gaming, perceived loss of control, craving, use of gaming to escape negative mood) which parents might have limited access to and therefore overlook or underestimate. Interviews were completed face-to-face by interviewers who had a college or university degree in health or education sciences and prior experience with conducting on average > 500 psychiatric interviews each. To assess interrater reliability 13% of the IGDI interviews (*n* = 88) were re-coded by raters blind to all information about the participants. The reliability of IGDI derived diagnosis was κ = 0.65 and the reliability for number of symptoms was ICC = 0.90.

#### Symptoms of Comorbid Disorders

Symptoms of ADHD (ICC = 0.90), oppositional defiant disorder (ODD, ICC = 0.91), conduct disorder (CD, ICC = 0.86), anxiety disorders (i.e., social phobia, specific phobias, separation anxiety disorder, general anxiety disorder, ICC = 0.87), and depressive disorders (major depression, dysthymia, ICC = 0.85) were assessed with the CAPA at age 8 and 10. Both parent and child were interviewed, and a symptom was considered present if reported by either parent or child. The aforementioned interrater reliabilities were obtained from re-codings by blinded coders of 15% of filmed interviews.

#### Predictors

*Demographic and family factors.* Parents provided demographic information and ratings of the family climate using the 12-item General Functioning Scale of the McMaster Family Assessment Device (α = 0.88) (Epstein et al. 1983), and on the degree of monitoring of the child (reversely scored, α = 0.59) and inconsistent discipline (α = 0.68) using 3-item scales from the Alabama Parenting Questionnaire (Shelton et al. 1996). As regards *child factors*, parents completed the Children’s Behavior Questionnaire (Rothbart et al. 2001) at the second assessment (age 6 years) which yields three global temperamental factors: Negative affect (24 items, α = 0.81), Surgency (25 items, α = 0.85), and Effortful control (26 items, α = 0.80). Children completed the Self-Description Questionnaire I (Marsh et al. 1984) to assess Global self-esteem (8 items, α = 0.84), and teachers completed the Emotion Regulation Checklist (Shields and Cicchetti 1997) (8 items, α = 0.78). Children’s intelligence was assessed with Wechsler’s abbreviated scales of intelligence (Wechsler 1999) using the Word comprehension and Matrices scales. Executive functioning was measured through the Global executive composite of the Behavior Rating Inventory of Executive Function™ completed by teachers (98 items, α = 0.98) (Isquith et al. 2004). To assess physical activity the children were instructed to wear an ActiGraph GT3X accelerometer (Manufacturing Technology Incorporated, Fort Walton Beach, FL, USA) for seven consecutive days, 24 h a day, taking it off only when bathing or showering. Only daytime activity (06:00–23:59) was included. We applied the Evenson et al. (2008) cut-off point of ≥2296 counts per minute to calculate the number of hours the child spent on moderate and vigorous physical activity (MVPA) each day. *Peer and social factors.* Teachers completed the Olweus Bully Victim Questionnaire (Olweus 1989) (5 items, α = 0.77) and also provided information on child social competence by means of the total score on the Social Skills Rating System (Gresham and Elliot 1990) (30 items, α = 0.94). Because previous findings on the effect of sports participation on IGD do not provide conclusive answers to whether such effects are due to (supposedly) increased physical activity or to the participation/social aspects of sports (Henchoz et al. 2016), parents reported the number of sessions the child trained with a sports team each week.

### Statistical Analysis

The importance of each symptom to the diagnosis of IGD was examined for sensitivity, specificity, and predictive value. The importance of each symptom to an underlying continuous IGD construct was evaluated by means of a one-factor exploratory analysis of binary items. To what extent the symptoms tap one or more underlying dimensions was assessed using exploratory factor analysis with oblique GEOMIN rotation. Age-8 predictors of age-10 IGD-symptoms were evaluated using linear regression, first unadjusted and then adjusted for gender and gaming at age 8. Latent IGD factors by means of confirmatory factor analysis were constructed to examine whether variables predicted different IGD factors differently when factor solution with more than one IGD construct were modelled. Finally, significant predictors were entered together in a multivariate regression analysis. To arrive at correct population estimates given oversampling, results were weighted with a factor corresponding to the number of children in the population in a particular stratum divided by the number of participants in that stratum (inverse probability weighting). Analyses were performed in Mplus 7.31 (Muthén and Muthén 1998-2015), applying a robust maximum likelihood estimator which is robust to deviations from normality, and missing data were handled according to a full information maximum likelihood procedure under the assumption that data was missing at random.

## Results

### Prevalence

The prevalence of IGD (5+ symptoms) was low (unweighted *n* = 14), though 6 times higher among boys than girls (Table 2). The prevalence of individual symptoms varied considerably, with some symptoms being fairly common, particularly tolerance, unsuccessful attempt to control, and use of Internet games to escape or relieve negative mood. Symptoms tapping negative consequences were much less frequent, though more evident in boys.

**Table 2:** Prevalence of symptoms of Internet gaming disorder among 10-year olds

	Percentage (95% CI)			OR gender (95% CI)
	Total (n = 740)	Males (*n* = 360)	Females (*n* = 380)	
DSM-5 symptoms of IGD^a^				
1. Preoccupation with Internet games	7.5 (5.3–9.5)	11.4 (7.7–15.2)	4.0 (1.8–6.1)	3.1 (1.6–6.2)
2. Withdrawal	2.1 (1.1–3.2)	2.7 (0.9–4.4)	1.7 (0.4–2.9)	1.6 (0.6–5.6)
3. Tolerance	22.6 (19.2–25.9)	28.4 (23.1–33.7)	17.4 (13.2–21.6)	1.9 (1.3–2.8)
4. Unsuccessful attempts to control	17.9 (14.8–21.0)	22.9 (18.0–27.9)	13.4 (9.7–17.1)	1.9 (1.3–3.0)
5. Loss of interest	2.5 (1.2–3.7)	4.1 (1.8–6.5)	1.0 (0.1–1.9)	4.4 (1.4–13.5)
6. Continued excessive use despite psychosocial problems	2.3 (1.0–3.6)	4.2 (1.7–6.8)	.6 (0.03–1.5)	7.4 (1.5–38.0)
7. Deception	4.4 (2.8–6.0)	5.0 (2.5–7.5)	3.8 (1.8–5.9)	1.3 (0.6–2.8)
8. Escape or relieve negative mood	15.0 (12.2–17.9)	18.5 (14.0–23.0)	12.0 (8.4–15.5)	1.7 (1.1–2.6)
9. Jeopardized or lost relationship or educational career opportunity	1.8 (0.7–2.9)	2.8 (0.8–4.8)	0.9 (0.0–1.7)	3.3 (1.00–11.0)
Number of symptoms^1)^	0.7 (0.6–0.8)	0.9 (0.8–1.1)	0.5 (0.4–0.6)	0.4 (0.2–0.6)
Internet gaming disorder diagnosis	1.7 (0.7–2.7)	3.0 (1.0–5.0)	0.5 (0.0–1.2)	6.0 (1.4–25.0)

^a^IGD = Internet Gaming Disorder

### Importance of Specific Symptoms

Almost all children with the IGD-diagnosis had symptoms of tolerance and unsuccessful attempts to control gaming (see “sensitivity” column, Table 3), but fewer than 10% with such symptoms received a diagnosis (see “positive-predictive-value” column (PPV), Table 3). Most meeting the diagnostic threshold had lost interest in previous activities and hobbies, used gaming to escape or relieve negative mood, and about half had deceived family members or others concerning their gaming. Relatedly, about half of the children who presented one of these three symptoms received a diagnosis. Withdrawal symptoms were rare among those with an IGD-diagnosis and few children with this symptom met diagnostic criteria. Moreover, about half with a diagnosis fulfilled the preoccupation criterion, though most with preoccupation did not receive a diagnosis.

**Table 3:** Relationships between Symptoms of Internet Gaming Disorder and Internet Gaming Disorder Diagnosis

	Sensitivity (%)	Specificity (%)	PPV (%)	NPV (%)
1. Preoccupation with Internet games	56.3	93.3	13.6	99.1
2. Withdrawal	17.7	98.0	12.8	98.5
3. Tolerance	93.8	78.4	7.5	99.9
4. Unsuccessful attempts to control	93.5	83.2	9.1	99.9
5. Loss of interest	62.2	98.7	56.0	99.0
6. Continued excessive use despite psychosocial problems	48.5	98.7	42.1	99.0
7. Deception	54.5	96.4	22.5	99.1
8. Escape or relieve negative mood	68.8	98.7	50.0	99.4
9. Jeopardized or lost relationship or educational career opportunity	31.3	98.7	31.3	98.7

Sensitivity = Percentage of diagnosed children having the symptom; Specificity = Percentage of children without diagnosis not having the symptom; PPV = Positive predictive value – Percentage of children with the symptom that receive a diagnosis, NPV = Negative predictive value – Percentage of children without the symptom that do not receive a diagnosis

### Dimensions of IGD

A one-factor factor analysis of IGD-symptoms revealed that loss of interest and continued excessive use despite psychosocial problems loaded highly, whereas withdrawal loaded moderately (Table 4). Exploratory factor analysis indicated that a two-factor solution fitted the data better than the a-priori one-factor model, Δχ^2^ = 20.60, *df* = 8, *p* = 0.008, with a 3-factor solution not providing a better fit than the 2-factor solution, Δχ^2^ = 8.57, *df* = 7, *p* = 0.29. Symptoms associated with heavy involvement in Internet gaming (i.e., preoccupation, withdrawal, tolerance, escape or relieve negative mood) loaded on one factor, with a second factor defined by symptoms tapping negative consequences of gaming (i.e., loss of interest, continued excessive use despite psychosocial problems, deception, jeopardizing or losing relationships or educational opportunities). The mean communality was satisfactory (MacCallum et al. 1999), albeit somewhat wide, attributable to preoccupation, withdrawal and deception. The two factors correlated moderately (*r* = 0.46).

**Table 4:** Standardized factor loading and communalities of Internet gaming disorder symptoms, exploratory factor analysis

	1-factor EFA			2-factor EFA	
			Heavy involvement	Negative conse-quences	
	Factor loading	h^2^	Factor loading	Factor loading	h^2^
1. Preoccupation with Internet games	0.59	0.35	0.54	0.14	0.37
2. Withdrawal	0.38	0.15	0.47	0.00	0.22
3. Tolerance	0.71	0.06	0.86	-0.04	0.71
4. Unsuccessful attempts to control	0.74	0.50	0.50	0.37	0.57
5. Loss of interest	0.98	0.96	0.28	0.83	0.98
6. Continued excessive use despite psychosocial problems	0.83	0.68	−0.10	0.99	0.92
7. Deception	0.56	0.31	0.26	0.42	0.34
8. Escape or relieve negative mood	0.70	0.49	0.61	0.18	0.51
9. Jeopardized or lost relationship or educational career opportunity	0.63	0.40	0.01	0.71	0.51
Average communality		0.45			0.57

h^2^ = Communality. Model fit for 1-factor EFA: *χ*^*2*^ = 43.77, *df* = 27, *p* = 0.02, RMSEA = 0.029 (95% CI: 0.011, 0.044), CFI = 0.970, TLI = 0.960, SRMR = 0.112. Model fit for 2-factor EFA: *χ*^*2*^ = 20.30, *df* = 19, *p* = 0.38, RMSEA = 0.010 (95% CI: 0.000, 0.034), CFI = 0.998, TLI = 0.966, SRMR = 0.067. Model fit for 3-factor EFA (factor loadings not shown above): *χ*^2^ = 11.47, *df* = 12, *p* = 0.49, RMSEA = 0.000 (95% CI: 0.000, 0.036), CFI = 1.000, TLI = 1.003, SRMR = 0.053

### Comorbidity

The number of IGD-symptoms correlated weakly with symptoms of ADHD (*r* = 0.08 [95% CI: 0.00–0.16]), ODD (*r* = 0.15 [0.07–0.24]), anxiety disorders (*r* = 0.13 [0.06–0.20]), and major depressive disorder (*r* = 0.12 [0.03–0.20]), but was unrelated to CD (*r* = 0.07 [−0.01–0.14]). The two IGD factors identified above were not differentially correlated with symptoms of these psychiatric disorders (*p >* 0.05).

### Predictors

The number of age-10 IGD-symptoms was regressed on age-8 socio-demographic, parenting/family and child factors in separate analyses for each predictor, with and without adjustment for gender and age-8 h per day of gaming (Table 5). At age 8 only two children played for more than 4 h per day and none for more than 5 h. Thus, there were likely no children who fulfilled the criteria for IGD at age 8. Among family factors, only higher parental education (adjusted for gender and age-8 gaming) predicted fewer symptoms at age 10. Among child factors, better social competence and emotion regulation predicted fewer IGD-symptoms, as did a higher involvement in organized sports, and Word comprehension score following adjustments for gender and age-8 gaming.

**Table 5:** Predictors at age 8 years of symptoms of Internet gaming disorder at age 10 years

Predictors at age 8 years	Unadjusted B (95% CI)	*P* value	Adjusted B (95% CI)	*P* value
Internet gaming time – hours per day	**0.31 (0.17–0.45)**	**<0.001**	**0.25 (0.10–0.40)**	**0.001**
Child gender	**0.42 (0.24–0.60)**	**<0.001**	**0.34 (0.16–0.53)**	**<0.001**
Informant parent’s education (1–11)	**−0.03 (−0.07–0.01)**	**0.11**	**−0.04 (−0.08--0.01)**	**0.016**
Parental job level (1–6)	−0.03 (−0.09–0.04)	0.43	−0.04 (−0.10–0.02)	0.16
Parental gross annual income (0–13)	−0.02 (−0.05–0.01)	0.11	−0.02 (−0.05–0.00)	0.09
Parents married or cohabitating	−0.07 (−0.30–0.16)	0.53	−0.12 (−0.34–0.12)	0.29
Poor family climate (1–4)	−0.03 (−0.16–0.51)	0.79	−0.06 (−0.25–0.13)	0.53
Parental poor monitoring (1–5)	0.06 (−0.08–0.20)	0.39	.01 (−0.13–0.15)	0.87
Parental inconsistent discipline (1–5)	0.05 (−0.07–0.18)	0.42	.00 (−0.12–0.13)	0.95
Child temperament – Negative affect (1–7)	−0.01 (−0.09–0.07)	0.79	−0.02 (−0.09–0.06)	0.70
Child temperament – Surgency (1–7)	0.00 (−0.07–0.07)	0.91	−0.01 (−0.08–0.06)	0.76
Child temperament – Effortful control (1–7)	−0.03 (−0.09–0.03)	0.32	−0.03 (−0.09–0.04)	0.42
Child self-esteem (8–40)	0.00 (−0.01–0.01)	0.54	−0.01 (−0.01–0.00)	0.18
Child social competence (0–90)	**−0.21 (−0.30--0.11)**	**<0.001**	**−0.19 (−0.29--0.09)**	**<0.001**
Child emotion regulation (1–4)	**−0.11 (−0.18--0.05)**	**0.001**	**−0.11 (−0.17--0.04)**	**0.001**
Child bullied	−0.02 (−0.05–0.01)	0.16	−0.06 (−0.13–0.01)	0.11
Child hours in moderate and vigorous physical activity per day	−0.02 (−0.07–0.04)	0.58	−0.05 (−0.10–0.01)	0.10
Child, number of training session in organized sports per week	**−0.10 (−0.17- -0.02)**	**0.017**	**−0.12 (−0.19 - -0.04)**	**0.003**
Child intelligence – word comprehension	**−0.01 (−0.02–0.00)**	**0.13**	**−0.01(−0.02–0.00)**	**0.04**
Child intelligence - matrices	0.00 (−0.01–0.01)	0.77	0.00 (−0.01–0.01)	0.63
Child executive function (0–146)	0.00 (0.00–0.00)	0.81	0.00 (0.00–0.01)	0.45
Child symptoms of ADHD (0–18)	0.05 (0.01–0.09)	0.012	0.04 (0.00–0.07)	0.07
Child symptoms of ODD (0–8)	0.08 (0.01–0.15)	0.03	0.05 (−0.02–0.13)	0.14
Child symptoms of CD (0–15)	−0.10 (−0.07–0.27)	0.24	0.04 (−0.13–0.21)	0.64
Child symptoms of major depressive disorder (0–9)	0.07 (−0.04–0.19)	0.20	0.05 (−0.06–0.16)	0.36
Child symptoms of anxiety disorders (0–21)	0.08 (0.00–0.16)	0.043	0.07 (−0.01–0.15)	0.066

Adjusted Bs are adjusted for gender and Internet gaming at age 8. Significant adjusted Bs in bold

A multivariate analysis with significant predictors was tested next. Because emotion regulation and social skills were highly correlated (*r* = 0.88), it was not possible to include both variables in the same model. This analysis showed previous gaming time, *B* = 0.27 [0.13–0.42] *p* < 0.001, social skills, *B* = −0.01 [−0.01–0.00], *p* = 0.007, and gender, *B* = 0.32 [0.13–0.51], *p* = 0.001, to uniquely contribute to the prediction of future IGD-symptoms, whereas sports participation was rendered insignificant, *B* = −0.06 [−0.16–0.02], *p* = 0.13), *R*^2^ = 0.08. Of note, the correlation between sports involvement and social skills was fairly high, *r* = 0.36, *p* < 0.001. If social skills was removed from the model, the seeming impact of sports participation became significant, *B* = −0.10 [−0.18 – −0.02], *p* = 0.029).

To gain further insight into possible mechanisms by which word comprehension and parental education might influence gaming behavior, we tested multiple mediation within a structural equation model in which social skills and sports participation were regressed on these two measures as, and symptoms of IGD were regressed on all of these measures, while controlling for gender and previous gaming time. Neither Word comprehension, *B* = 0.00 [−0.001–0.01] nor parental education, *B* = −0.03 [−0.07–0.01] had any direct effect on IGD symptoms. Notably, their respective negative effects on IGD-symptoms were mediated via social competence, with the following indirect effects: parental education, *B* = −0.01, *p* = 0.035, Word comprehension, *B* = −0.01, *p* = 0.01. Recall that sports participation was not predictive of IGD symptoms when social skills were adjusted for. Hence, sports participation could not act as a mediator. Replacing social skills with emotion regulation yielded similar results. The symptom “using Internet games to relieve or escape negative mood” could be considered a strategy for emotion regulation. We therefore constructed a measure of IGD symptoms deleting this item. Even this abbreviated 8-item IGD measure was predicted by emotion regulation, unadjusted *B* = −0.09, [−0.15- -0.03] and adjusted for previous gaming and gender, *B* = −0.09, [−0.14- −0.04].

Because the factor analytic findings revealed a Heavy involvement and a Negative consequences factor, it is possible that different variables predicted the two factors. To test this possibility a model assuming identical predictors of these two latent constructs was compared to a model in which predictors were free to vary, using a Wald test. To avoid differential scaling of the latent variables to influence the results, the residuals of the two latent factors were both set to1. None of the age-8 variables and age-6 temperament were differentially predictive of the two constructs. However, anxiety symptoms at age 10 correlated with heavy involvement, *r* = 0.22, *p* > 0.001, but not with Negative consequences, *r* = −0.02, *p* = 0.82, Wald = 8.18, *p* = 0.004. Symptoms of other disorders were not differentially correlated with the two IGD factors.

## Discussion

This is the first study to examine, using a diagnostic interview, the prevalence of IGD and individual symptoms as proposed in the DSM-5, how symptoms relate to the disorder, comorbidities and predictors of symptoms of IGD in the community. We found the prevalence of IGD to be 1.7% in Norwegian 10-year olds. The nine proposed symptoms seem to define two correlated constructs, one reflecting heavy gaming involvement and one negative consequences. Symptoms of IGD were only weakly correlated with symptoms of other psychiatric disorders. Among the hypothesized predictors, low social competence and poor emotion regulation at age 8, multivariately forecasted more age-10 IGD symptoms, mediating effects of limited parental education and word comprehension. Symptoms of IGD were also predicted by little involvement in organized sports, but this effect vanished once social competence was included in the prediction model.

### Prevalence, Dimensions, and Importance of Specific Symptoms

Previously reported prevalence rates of IGD range from 1.2% (Rehbein et al. 2015) to 8.5% (Gentile 2009). As youth continue to increase their Internet gaming into adolescence, we should expect the rates of IGD to increase as well, though a decline might emerge when the adolescents enter adulthood (Rehbein et al. 2016). Applying the DSM-5 criteria and using a diagnostic interview, the prevalence discerned herein is at the lower end. Whether the proposed symptoms actually capture a disorder or merely heavy involvement in Internet gaming is much disputed (Griffiths et al. 2016; Kardefelt-Winther 2015; Petry et al. 2014). Two correlated dimensions underlying IGD symptoms, strong gaming involvement and negative gaming consequences, resemble previous results (Brunborg et al. 2015; Charlton and Danforth 2007). Most of the symptoms tapping negative consequences had fairly high sensitivity, specificity, and PPV. As regards one of these symptoms, loss of interest in previous activities and hobbies, some have argued that there is nothing inherently pathological in switching interest from previous interests to Internet gaming (Griffiths et al. 2016). Notably, losing interest in previous interests was not normative (2.5% prevalence). Among those who did, however, more than half received an IGD diagnosis and almost two thirds of those with IGD affirmed this symptom; moreover, it loaded perfectly on a continuous IGD factor. Thus, although it may seem harmless to give up previous interests to gaming, those with IGD typically do so-- and those who do so typically have IGD; this result closely resembles that of a questionnaire study of German adolescents (Rehbein et al. 2015). Thus, although infrequent, loss of interest may be a core feature of IGD across ages.

To receive an IGD diagnosis five symptoms are needed. There are no strong arguments for why a cut-off of five was suggested in the DSM-5, understandably so, since such information was lacking. However, before the number of symptoms is decided upon, the appropriate content of IGD-symptoms should be investigated further since symptoms have different prevalence and may be of unequal importance to the IGD construct. Because the majority of the heavy involvement symptoms are prevalent, many individuals who cross the diagnostic threshold will likely manifest all or many of the four heavy-involvement symptoms, but only one or a few relating to negative consequences. Two of the heavy involvement symptoms, tolerance and unsuccessful attempt to control gaming, had low PPV, which suggests that they were unspecific to those with the disorder. This accords with the theoretical argument that tolerance does not make sense in the context of Internet gaming (Kardefelt-Winther 2015). Empirically, however, other investigators using questionnaires have reported tolerance to be central to a continuous IGD construct as well the disorder (Lemmens et al. 2015; Rehbein et al. 2015). It should also be recognized that the TESS participants were young and likely to increase their Internet gaming as they age; increased gaming should thus be expected and not pathologized. Hence, the centrality of the tolerance criterion may vary by age.

Gaming to relieve or escape negative moods was fairly prevalent (15%). Indeed, gaming to relieve negative moods is typical of those with heavy involvement (Griffiths et al. 2016), including those who do not necessarily have the disorder. Even so, the PPV was among the highest (50%); the specificity was high; and the item also loaded highly on the one-factor IGD dimension. Thus, empirically, the escape symptom seems to be central to IGD in this population.

As regards withdrawal, its sensitivity was very low; its PPV was low; and it loaded moderately on an underlying IGD factor. Contrary to previous factor-analytic (Lemmens et al. 2015) and inference-tree (Rehbein et al. 2015) findings, then, withdrawal does not seem to define IGD in the present population when a clinical interview was applied. In sum, if our findings are replicated, the content, number of and balance between heavy involvement and negative consequences symptoms should be considered in future revisions of the IGD diagnosis.

### Comorbidity

Finding symptoms of IGD to correlate (somewhat) positively with symptoms of most common psychiatric disorders in youth is in line with previous research (Kuss et al. 2014). Notably, though, associations were considerably weaker than between symptoms of other disorders (Costello et al. 2003; Wichstrøm et al. 2018), and no specific pattern of associations was seen. Thus, the data presented herein does suggest that IGD belongs to neither the internalizing nor externalizing spectrum. The presence of comorbidity does not speak directly to whether IGD should be considered a disorder or not. However, the weak associations with symptoms of more established psychiatric disorders indicate that the latter are not likely predictors or consequences of IGD symptoms, at least in this young age group.

### Correlates and Predictors

Two prior prospective studies identified poor social competence and emotion-regulation skills as predictive of later symptoms of pathological video-gaming using self-completed questionnaires (Gentile et al. 2011; Liau et al. 2015). We replicated these Singaporian findings using a diagnostic interview. Possibly due to allowing more time for deliberate response and not giving away non-verbal cues of social insecurity or appearance, children who are less socially competent in face-to-face interaction may prefer the Internet for social interaction (Kowert and Oldmeadow 2013). Whether poor social competence is indeed a risk factor for IGD or for simply spending much time on the Internet awaits future research. Like others, we find that emotion-regulation deficits predict IGD symptoms, but further show that this also holds when IGD is examined by a diagnostic interview and when “using gaming to reduce negative mood” is not an included symptom. Finally, low parental education and less child word comprehension emerged as predictors of IGD symptoms, seemingly through reduced child social competencies.

The gender difference in gaming and IGD in particular has been known for a long time. What is it about boys that make them more susceptible to IGD than girls? One may speculate that it is because they simply game more than girls, or that other risk factors such as poor social competence, and high levels of aggression and impulsiveness are more prevalent in boys (Rehbein et al. 2010). However, even when such trait-like characteristics were taken into account, boys evinced more IGD-symptoms than girls in the present study. This increase risk of becoming addicted in young males is seen in a range of areas, including gambling (Dowling et al. 2017) and alcohol use (Rohde et al. 2001). Thus, common explanations for all addictions should be considered and investigated. However, one should not overlook the possibility that gaming-specific factors such as boys’ stronger preference for games that may be particularly addictive such as role-playing and first-person shooting games may explain the gender difference, as one study suggested (Rehbein et al. 2016). Relatedly, games may be constructed to be more attractive to boys.

We failed to identify several correlates and predictors of IGD symptoms and related Internet addiction, as well as of pathological video gaming, that have emerged in prior cross-sectional (e.g., Ko et al. 2012; Kuss et al. 2014; Li et al. 2014) and prospective (e.g., Henchoz et al. 2016; Liau et al. 2015) studies, including family climate, parenting, child temperament, symptoms of psychiatric disorders, physical activity/sport participation, victimization, and self-esteem. Conceivably, this is due to the fact that most prior work used self-completed questionnaires to assess all construct, thus raising the possibility that reported associations are inflated due to common methods. Such inflation seems less likely in the work reported herein given that we relied on diverse methods and information sources. Moreover, the lack of adjustment for likely confounders may have inflated some previous estimates. To illustrate, as in a Swiss study (Henchoz et al. 2016) we found sports participation to predict future IGD, over and above previous gaming, gender, and objectively measured level of moderate and vigorous physical activity. However, unlike the prior investigation, once we adjusted for the higher social competence of those involved in sports, the effect of sports vanished.

### Limitations

We must acknowledge detection of only a few IGD cases, resulting in somewhat large confidence intervals for the prevalence estimate, 0.7% to 2.7%. This uncertainty should also be considered in the context of moderate reliability of the IGD disorder, which might be due to the low prevalence of IGD in the population and thus uncertainty in the reliability sub-sample. Second, we investigated 10-year olds in one Norwegian community. Prevalence may increase as children age--because gaming is highest in 12–14 year olds (Gentile 2009; Medietilsynet 2015). Although it has been difficult to discern country differences in prevalence because of the wide variety of assessment instruments and definitions of IGD employed (Petry et al. 2015), the prevalence documented herein may be different in other countries, and in other populations such as those seeking help for mental health problems. The fact that all Norwegian children have access to the Internet should also be considered when generalizing the present findings. Nonetheless, the rate of Internet gaming was similar to that found in Norwegian (Medietilsynet 2015), German (Rehbein and Baier 2013), Spanish (Pujol et al. 2016), and British (Przybylski 2014) preadolescent samples, indicating generalizability of results at least to European youth. That said, the rates of other mental health problems are generally lower among Norwegian children than in many other Western countries (Heiervang et al. 2007; Wichstrøm et al. 2012; Wichstrøm et al. 2018), suggesting that generalization to other populations should be tentative. We relied on child-reports to determine IGD because many of the IGD symptoms are likely difficult to reliably and validly assess for others (e.g., anticipation, gaming to escape negative mood) or youth may actively conceal their gaming from parents. Moreover, it is possible that youth lose track of for how long they game once they are absorbed in gaming, or that they consciously underreport gaming time or other symptoms when queried for social desirability reasons. Even though parents may not be in an expert position to rate all symptoms, they could certainly report on some of them, and this lack of parental information is a limitation, possibly deflating the number of symptoms and the rate of disorder. Even though the over-determination was good in our 2-factor solution, the communalities evinced some width, implying that it should be interpreted with caution as we cannot rule out the possibility that an even higher number of participants might have revealed different patterns. We were only able to adjust for time spent on gaming at age 8 and not IGD-symptoms when examining the predictors of age 10 IGD-symptoms, though very few children played for an extended time at age 8 and thus few were expected to IGD-symptoms. Moreover, some predictors had only modest reliability (e.g., parental monitoring and inconsistent discipline) and this may have deflated associations with symptoms of IGD. Finally, we interviewed only the youth on IGD. As previously mentioned we had strong reasons to believe that parents might underestimate the amount of gaming and the prevalence of IGD symptoms due to a lack of information. However, we cannot discount the possibility that the youth might also underreport gaming and IGD symptoms, in part for social desirability reasons, and that at least some parents might have provided information that is more correct.

### Conclusions

DSM-5-defined IGD is already present in some 10-year olds, with a strong male preponderance. The nine proposed symptoms tap two underlying factors, heavy involvement in gaming which proved quite common, and negative gaming consequences which was rather rare. Two of the common heavy-involvement symptoms, tolerance and unsuccessful attempts to control gaming, were not very specific to IGD. The withdrawal symptom was weakly related to IGD, both as a diagnosis and as continuous construct. It is important to investigate the content and relevance of these three criteria further. Symptoms of IGD correlate modestly with symptoms of other psychiatric disorders. Although a wide range of potential child, family and demographic predictors were considered, only low social competence and poor emotion regulation skills predict more IGD-symptoms.

## Electronic Supplementary Material

ESM 1 (DOCX 26 kb)
