# Supplementary material for: Symptoms of Internet Gaming Disorder in Youth: Predictors and Comorbidity
Source: J Abnorm Child Psychol. 2018 Apr 5;47(1):71–83. doi: 10.1007/s10802-018-0422-x (PMC6329732; doi:10.1007/s10802-018-0422-x)
Supplement: Supplementary file 1 — (DOCX 26 kb) [file 10802_2018_422_MOESM1_ESM.docx]

**eTable1.** Operationalization of DSM-5 Symptoms of Internet gaming disorder and interrater reliabilities of symptoms and disorder (kappa or intraclass correlation)

| **Symptom** | **κ** | **Description and 12 month frequency or duration each day** |
| --- | --- | --- |
| 1. Preoccupation | 0.87 | (i) Gaming duration: 30+ hours per week |
|  |  | or (ii) Thinks about previous games: twice a day |
|  |  | or (iii) Anticipates playing the next game: twice a day |
|  |  | or (iv) Planning the day to enable for extensive gaming: once a day |
|  |  | or (v) Discuss or seek out instructions on how to game: twice a day |
| 2. Withdrawal | 0.71 | Anxiety, worry, anger, irritability, sadness, or sleep problems following absence of gaming for at least 24 hours after a period of gaming that lasted at least 3 days. |
| 3. Tolerance | 0.86 | Significant increase in the amount of gaming or a wish for significantly more gaming |
| 4. Unsuccessful attempts to control | 0.67 | Unsuccessful attempts to reduce the amount of gaming or to stop gaming, initiated by the person or by parents |
| 5. Loss of interest | 1.00 | Loss or substantial reduction in the interest in previous hobbies, entertainment or activities as a result of gaming |
| 6. Continued excessive use despite psychosocial problems | 0.62 | The person acknowledges that gaming is at the expense of psychological or social functioning, yet does not reduce or stop gaming |
| 7. Deception | 0.96 | Deceived family members or others concerning the amount of time spent on gaming |
| 8. Escape or relieve negative mood | 0.91 | Gaming to escape from or reduce negative emotions such as guilt, anxiety, low self-esteem, perceived helplessness, or loneliness |
| 9. Jeopardized or lost relationship or educational career opportunity | 0.92 | Risked losing or has lost a significant social relation, work, important activity, schooling – including substantially lower school results due to gaming |
| Sum of symptoms 1-9 | 0.90¶ |  |
| Internet gaming disorder diagnosis | 0.65 |  |

Note: ¶=Intraclass correlation. To assess interrater reliability 13% of the IGDI interviews (n=88) were re-coded by raters blind to all information about the participants.

**eText 1.** The Internet Gaming Disorder Interview (IGDI)

**Introduction**

The IGDI is an instrument to measure Internet gaming disorder and its symptoms as outlined in the Addendum to DSM-5. IGDI is an interviewer-based interview. Hence, the interviewer should decide whether the symptom is present or not at the specified levels of severity and intensity, not solely the interviewee. To accomplish this, the mandatory and optional questions should be asked. In the case that the interviewer cannot decide on whether the symptom is present or absent from the responses to the mandatory and optional questions, the interviewer should continue to probe until she or he can decide, or conclude that she or he cannot decide. For details on the style of interviewing and coding rules, see the glossary to the Child and Adolescent Psychiatric Assessment (CAPA) <http://devepi.duhs.duke.edu/eMeasures/CAPA%20glossary.pdf> and the glossary for the Preschool Age Psychiatric Assessment (PAPA) <http://devepi.duhs.duke.edu/eMeasures/PAPA%20Glossary.pdf>. If the interviewee has difficulty remembering or stating exact frequencies, use the previous week and work through each day. If necessary divide the day into sections (e.g., morning before going to school, after dinner - but before soccer practice, at night). Ask if this was a typical week. If not, clarify deviations or development during the past 12 months.

Optional questions are in italics.

**Definitions and Operationalization**

Internet gaming refers to gaming activities on the Internet, not other forms of Internet use. Commonly, more than one player is involved, but this is not a requirement. Games played offline on computers and similar platforms (e.g., smartphones, Ipads, tablets, Nintendo, Playstation, Xbox) should be included. Do not include board games. Exclude Internet use in relation to schoolwork or paid jobs, social media, music, videos, file sharing, or use of sexual material.

*In the following, only symptoms present during the past 12 months are coded. Frequencies of symptoms or behaviors are converted from 12 months to a 3-month duration (as in the CAPA). Please note that there are exceptions to this rule where symptoms or behavior are coded if they have ever been present.*

As for most other disorders described in the DSM-5 the decision to code presence or absence of a specific symptom rests on several qualitative decisions where few direct guidelines can be derived from the DSM-5 text. The reasoning behind the decisions made to code the respective symptoms is described below.

**Decisions concerning preoccupation with Internet games.** In the DSM-5 diagnostic criteria, three examples of preoccupation are mentioned: (i) thinking about previous gaming, (ii) anticipates playing the next game, and (iii) that Internet gaming becomes the dominant activity in daily life. The duration and frequency of these cognitive and behavioral activities are not specified. This is therefore left to the clinician/interviewer/researcher. We have chosen a conservative approach, thus in keeping with the notion that Internet gaming should be the dominant activity, and such thinking (in i and ii) should therefore occur every day for the past 12 months, and at least twice a day. However, because Internet gaming takes much time, time must be allotted to gaming. In order to become the dominating activity the person has to organize his or her daily activities so that gaming can takes place. Making such plans therefore becomes one way the ‘dominating activity’ shows. However, once a plan has been set, the person does not necessarily has to rework it, hence such a plan need only to be made once each day, every day for the past 12 months. For Internet gaming to become the dominating activity, such games have to be played for an extended period. The DSM-5 specifies *“… at least 30 hours per week*.” This 30 hour per week is therefore set as the lower limit in IGDI. Preoccupation may take different forms with different addictions. The DSM-5 lists thinking and anticipation, i.e. cognitive activity, as signs of preoccupation. Because Internet gaming is also a skill, many gamers spend much time not just gaming /practicing but also seek out information and tips on how to be more successful or advance in the game, typically watching streamed or downloaded videos from expert players or discussing games or strategies with friends or fellow gamers. This behavioral/informational sign of preoccupation has been overlooked in the DSM-5, but included in the IGDI.

**1A. Preoccupation with Internet games: Internet gaming is the dominating activity in daily life – excessive use**

How often do you game? *Every day? How much during a month?*

Number of days in a 3-month period: __ __

How many hours per day do you game, when you are gaming? And, how many days do you game in a week?

|  | Hours per day when gaming | Minutes per day when gaming | Number of days per week |
| --- | --- | --- | --- |
| **During weekdays** |  |  |  |
| Do you game in the **morning** before going to school?  *How long? How often?* |  |  |  |
| Do you game at **school**?  *How long? How often?* |  |  |  |
| Do you game in the **afternoon** after school is finished, but before dinner?  *How long? How often?* |  |  |  |
| Do you game in the evening, after dinner?  *How long? How often?* |  |  |  |
| Do you game at night?  *How long? How often?* |  |  |  |
| **During weekends (Saturday and Sunday)** |  |  |  |
| Do you game in the morning on weekends or holidays?  *How long? How often?* |  |  |  |
| Do you game during the day, before dinner?  *How long? How often?* |  |  |  |
| What about in the evening?  *How long? How often?* |  |  |  |
| And at night?  *How long? How often?* |  |  |  |

*If the interviewee is using more than 1 hour per day gaming:* When did you start using about the amount of time you are using now on gaming?

Day __ __ Month __ __ Year __ __

**1B. Preoccupation with Internet games: Internet gaming is the dominating activity in daily life – thinks about previous games**

Do you ever think about games that you have played? Yes/No

*Every day? How about each month? How often?* Number of days in a 3-month period: __ __ __

When you start thinking about this, how many times a day did you do that?

Times per day: __ __ __

When did you start thinking about previously played games?

Day __ __ Month __ __ Year __ __

**1C. Preoccupation with Internet games: Internet gaming is the dominating activity in daily life – anticipated playing the next game**

Do you ever think about games that you will play? Yes/No

*Every day? How about each month? How often?* Number of days in a 3-month period: __ __ __

When you are thinking about this, how many times a day do you do that? Times per day: __ __ __

When did you start thinking about the games you would be playing?

Day __ __ Month __ __ Year __ __

**1D. Preoccupation with Internet games: Internet gaming is the dominating activity in daily life – planning the day so as to be able to game**

Do you plan your day so that you have time to game? Yes/No

*Every day? How about each month? How often?* Number of days in a 3-month period: __ __ __

When you are thinking about these plans, how many times a day do you do that each day?

Times per day: __ __ __

When did you start planning your days in order to have time to game?

Day __ __ Month __ __ Year __ __

**1E. Preoccupation with Internet games: Internet gaming is the dominating activity in daily life – learning and talking about games**

Does it ever happen that you inquire (e.g., search for or watch You Tube videos) about new games or games that you are playing? Do you talk to your friends or others about the games you have played, are planning to play, or how to play a particular game? Yes/No

*Every day? How about each month? How often?* Number of days in a 3-month period: __ __ __

How many times a day do you inquire or talk about playing each day?

Times per day: __ __ __

When did you start to inquire or talk, in such a fashion, about gaming?

Day __ __ Month __ __ Year __ __

**2. Withdrawal symptoms when Internet gaming is taken away (abstinence)**

*To count as a withdrawal symptom, the symptom(s) must have appeared within 24 hours after withdrawal of the game (or reduced amount of gaming) after a period of heavy gaming (that lasted at least 3 days)*

Have you ever not gamed for a whole day, for 24 hours?

What happened when you stopped gaming?

*Has it ever happened that you became restless (e.g., it was difficult to sit still)?*

*Have you ever become anxious or worried? Angry or irritable? Felt sad? Had a lot of negative thoughts? Difficulties to fall asleep?* Yes/No

Number of days in a 3-month period: __ __ __

When was the first time you experienced this when stopping to game?

Day __ __ Month __ __ Year __ __

**3. Tolerance -- the need to spend increasing amounts of time engaged in Internet games**

*A marked need to increase the amount of gaming as compared to previous gaming, thus a much sharper increase in the amount of time spent on gaming than is to be expected from age typical increase in gaming.*

Do you game more now than before? Do you game for much longer periods now than before?

*Do you game during more periods of the day now than you used to (e.g., in the morning, at school, after bed time)?*

*If there were no house rules as to how much you were allowed to game, would you game more than you do to today? How much? Would you continue to do so?*

*Do you want to game more than you do, or used to? – How much more?* Yes/No

When did you start to game more or want to game more?

Day __ __ Month __ __ Year __ __

**4A. Unsuccessful attempts to control participation in Internet games – own attempts**

*Actual attempts, initiated by the interviewee, to reduce or stop gaming (i.e., not just wanting to) that lasted for at least 24 hours during the previous 12 months, but did not succeed in permanent reduction in gaming.*

Have you ever really tried to game less? Yes/No

*Why did you do this?*

*How many times have you tried?*

*When was the last time?*

*What happened?*

Number of times in a 3-month period: __ __ __

When was the first time you tried to cut down or stop gaming?

Day __ __ Month __ __ Year __ __

**4B. Unsuccessful attempts to control participation in Internet games – parent initiated attempts**

*Parents have, during the previous 12 months, initiated reduction (demanded reduction, prohibited gaming, removed or disconnected router, PC, smartphone, or other gaming equipment) with the intention to achieve the youth abstaining from gaming for at least 24 hours or a permanent reduction in gaming, but did not succeed in such abstinence or permanent reduction.*

Have your parents or others tried to reduce how much you game, e.g. set up rules, prohibited gaming, taken your PC, the Internet connection etc.? Yes/No

*For how long?*

*Why did they do this?*

*What happened?*

*How many times have they done this?*

*When was the last time?*

*What about the last 3 months?*

Number of times in a 3-month period: __ __ __

When was the first time they tried to reduce your gaming?

Day __ __ Month __ __ Year __ __

**5. Loss of interest in previous hobbies and entertainment**

*A loss of, or substantial reduction, in interest in previous hobbies or other types of entertainment or activities, as a result of Internet gaming. Do not include age typical changes/reduction in activities or relationships.*

Have you stopped doing things you liked before **because of gaming**? Yes/No

Or done much less of it?

*Like sports or hobbies?*

*What about reading?*

*Listen to music?*

*What about being with friends (not on the Internet)?*

*What about parents or siblings, do you spend less time with them now than before?*

*Why did you stop, or spend less time?*

*Why is it less now?*

*Have you given up something else?*

When did this start?

Day __ __ Month __ __ Year __ __

**6. Continued excessive use of Internet games despite knowledge of psychosocial problems.**

*The interviewee must have some acknowledgement that his/her gaming leads to problems, in areas such as homework, school, attention, grades or school performance, mood, sleep, relationships to others (e.g., more quarrels with parents, less time devoted to friends or siblings), time spent on hobbies, sports, eating, etc.*

Have you done less of something or has your gaming lead to problems?

Have you spent less time with others? Yes/No

*What about homework?*

*Follow the instruction in class?*

*Sleep, or when you go to bed, or waking up in the night and leaving the bed?*

*Been with friends?*

*Do things with you parents or siblings?*

*What about your mood?*

*Are you just as rested and attentive as before?*

*What about arguing with others, parents, siblings?*

*Was this different when you gamed less or not at all?*

When did you notice this? When did it start?

Day __ __ Month __ __ Year __ __

**7. Has deceived family members, therapists, or others regarding the amount of Internet gaming.**

*Has deceived family members or others with respect to how much he/she games.*

Have you ever tried to hide your gaming? Yes/No

*Do your parents know how much you game?*

*Do you game for longer hours or more often than you are allowed to?*

*At other hours than you are allowed to?*

*What about after school?*

*At night?*

*What would your parents say if they knew how much you did game?*

When did this start?

Day __ __ Month __ __ Year __ __

**8. Use of Internet games to escape or relieve a negative mood.**

*Use of Internet gaming to flee from or reduce negative emotions (e.g., perceived helplessness, guilt, anxiety, low self-worth, loneliness).*

Do you ever game to stop thinking about difficult things in your life?

If you did not game, how would it be for you?

Would there be things that were difficult, such as being sad or depressed or anxious? Yes/No

*Does gaming help, e.g. that you do not get such feelings or thoughts when you game?*

When did you realize that gaming helped in such a way?

Day __ __ Month __ __ Year __ __

**9. Has jeopardized or lost a significant relationship or educational career opportunity**

*Has risked losing, or lost an important relationship or work; has considerably less school attendance or have gotten substantially poorer school results due to gaming.*

Have you stopped being with a friend or more friends, or been very much less with them **because of your gaming**?

Has your gaming lead to much poorer schoolwork or your results at school?

*Would you have done much more homework if you had gamed less? Did you use to?*

*What about friends, would you have been more with them if you gamed less?*

*What about sports?*

*Or your parents or siblings?*

When did this start?

Day __ __ Month __ __ Year __ __
